# Transcriptomic and Functional Analyses Reveal the Different Roles of Vitamins C, E, and K in Regulating Viral Infections in Maize

**DOI:** 10.3390/ijms24098012

**Published:** 2023-04-28

**Authors:** Kaiqiang Hao, Miaoren Yang, Yakun Cui, Zhiyuan Jiao, Xinran Gao, Zhichao Du, Zhiping Wang, Mengnan An, Zihao Xia, Yuanhua Wu

**Affiliations:** 1 College of Plant Protection, Shenyang Agricultural University, Shenyang 110866, China; 2 Institute of Food Crops, Jiangsu Academy of Agricultural Sciences, Nanjing 210014, China; 3 State Kay Laboratory of Agrobiotechnology and Key Laboratory of Pest Monitoring and Green Management-MOA, Department of Plant Pathology, China Agricultural University, Beijing 100193, China

**Keywords:** maize lethal necrosis (MLN), maize chlorotic mottle virus (MCMV), sugarcane mosaic virus (SCMV), differentially expressed isoforms (DEIs), vitamins C, E, and K, pathogenesis-related (*PR*) genes

## Abstract

Maize lethal necrosis (MLN), one of the most important maize viral diseases, is caused by maize chlorotic mottle virus (MCMV) infection in combination with a potyvirid, such as sugarcane mosaic virus (SCMV). However, the resistance mechanism of maize to MLN remains largely unknown. In this study, we obtained isoform expression profiles of maize after SCMV and MCMV single and synergistic infection (S + M) via comparative analysis of SMRT- and Illumina-based RNA sequencing. A total of 15,508, 7567, and 2378 differentially expressed isoforms (DEIs) were identified in S + M, MCMV, and SCMV libraries, which were primarily involved in photosynthesis, reactive oxygen species (ROS) scavenging, and some pathways related to disease resistance. The results of virus-induced gene silencing (VIGS) assays revealed that silencing of a vitamin C biosynthesis-related gene, *ZmGalDH* or *ZmAPX1*, promoted viral infections, while silencing *ZmTAT* or *ZmNQO1*, the gene involved in vitamin E or K biosynthesis, inhibited MCMV and S + M infections, likely by regulating the expressions of pathogenesis-related (*PR*) genes. Moreover, the relationship between viral infections and expression of the above four genes in ten maize inbred lines was determined. We further demonstrated that the exogenous application of vitamin C could effectively suppress viral infections, while vitamins E and K promoted MCMV infection. These findings provide novel insights into the gene regulatory networks of maize in response to MLN, and the roles of vitamins C, E, and K in conditioning viral infections in maize.

## 1. Introduction

Since the discovery of maize chlorotic mottle virus (MCMV) in the early 1970s, an emerging and rapidly spreading plant disease, maize lethal necrosis (MLN) has threatened maize production in many regions, especially in Africa [1,2]. MLN is caused by synergistic infection of MCMV and one of several viruses from the *Potyviridae*, such as sugarcane mosaic virus (SCMV) [1,2]. The relationship between maize and SCMV infection has been extensively studied, primarily in terms of protein–protein interactions, the changes in maize protein and microRNA (miRNA) profiles in response to SCMV infection [3,4,5,6]. Maize resistance to SCMV infection has been linked to two major quantitative trait loci (QTLs), *Scmv1* and *Scmv2*, which have been identified in diverse independent mapping populations [7,8]. However, the genetic basis of maize plant tolerance/resistance to MCMV/MLN is poorly understood [2]. Numerous QTLs associated with resistance to MLN across maize chromosomes in multiple mapping populations have been identified, while no maize inbred lines show significant resistance to MLN [2,9,10]. Moreover, several studies have been conducted to elucidate how maize responds to MCMV/MLN infection [11,12,13,14]. For the control of MLN in maize, novel methods for identifying effective resistance genes are urgently needed.

RNA sequencing (RNA-Seq) is the most commonly used method for determining the sequences of all RNA transcripts in a given specimen. However, short-read sequences produced by RNA-Seq may generate low-quality transcripts and lead to incorrect annotations, thus limiting their utility for discovering alternatively spliced isoforms [15]. Recently, single-molecule real-time (SMRT) long-read sequencing technology based on Pacific BioSciences (PacBio) has been developed and utilized, which provides complete sequence information of cDNA molecules without the need for further assembly [16,17]. Presently, a combination of SMRT isoform sequencing (Iso-Seq) and RNA-Seq is currently widely used to identify genes involved in responses to various stresses [16,18,19].

During plant–virus interactions, defense responses can be induced by the accumulation of reactive oxygen species (ROS) [20]. ROS function as diffusible signals that induce antioxidant and pathogenesis-related defense responses in adjacent plant cells. When ROS are present at high concentrations, oxidative stress and programmed cell death (PCD) occur in plants, limiting the spread of viruses between cells. In NN tobacco (a cultivar of *Nicotiana tabacum* containing the *N* gene), a burst of reactive oxygen intermediates (ROIs) occurs as early as 10 min after tobacco mosaic virus (TMV) infection, ultimately producing a hypersensitive response (HR) in tobacco leaves that limits TMV cell movement [21]. Therefore, we speculated that ROS play important roles during MCMV and SCMV infection in maize. Vitamins, especially vitamin C, vitamin E, and vitamin K, play crucial roles in ROS hemostasis in plants [22,23,24], while their roles in regulating viral infections in maize remain to be clarified.

Vitamin C (L-ascorbic acid; AsA) is a crucial secondary metabolite in plants that acts as an antioxidant and cell signaling modulator in a wide range of vital physiological processes, such as cell division, growth regulation, and senescence [22,25]. In a recent study, AsA production was shown to be reduced in *TaVTC2* knockdown plants (*TaVTC2*-RNAi), resulting in ROS burst and wheat yellow mosaic virus (WYMV) resistance in wheat leaves [26]. In plants, L-galactose dehydrogenase (GalDH) is a key enzyme in the biosynthesis of AsA [27]. The activity of GalDH was only 30% of the normal level in *Arabidopsis thaliana* lines transformed with antisense *GalDH* constructs, which had lower leaf AsA concentrations when grown under high light [28]. Ascorbate peroxidase (APX), an enzyme that utilizes AsA as an electron donor, is a reducing substrate-dependent peroxidase involved in intracellular hydrogen peroxide (H_2_O_2_) removal [29,30]. Both AsA and APX are reportedly involved in viral infections. For example, radish accumulates AsA to improve its defenses against turnip mosaic virus (TuMV) by increasing jasmonic acid (JA) levels [31]. APX activity was shown to be increased significantly in mungbean yellow mosaic India virus (MYMIV)-resistant *Vigna mungo* plants [32].

In plants, α-tocopherol is a major vitamin E component found in chloroplast envelopes, thylakoids, and plastoglobuli [23]. As an antioxidant, α-tocopherol can inactivate ROS generated by photosynthesis and has been shown to reduce the extent of lipid peroxidation in leaves and seeds [33,34]. Moreover, it has been revealed that α-tocopherols can increase the tolerance of tomatoes to TMV infection [35]. Tyrosine aminotransferase (TAT), which catalyzes the transamination from tyrosine to *p*-hydroxyphenylpyruvate, is the first enzyme in a pathway from homogentisic acid to tocopherols [36,37].

Vitamin K (1,2-methyl-3-phytyl-1,4-naphtoquinone), also known as phylloquinone (PHQ), is necessary for the normal functioning of animals and plants [24]. Vitamin K shows potent antioxidant activity in solvents and isolated membranes and functions in redox reactions at the plasma membrane of plants [38]. Arabidopsis mutants defective in vitamin K biosynthesis exhibit severe reductions in photosystem I (PSI) activity and chlorophyll content [39]. It has been reported that NAD(P)H quinone oxidoreductase (NQO1) has conserved functions conservatively in animals, plants, and even microorganisms, maintaining the active forms of several endogenous antioxidants, especially vitamin K [40,41].

In this study, we adopted PacBio SMRT Iso-Seq combined with RNA-Seq to identify specific genes and pathways involved in resistance to SCMV, MCMV, and synergistic infection of SCMV and MCMV (S + M) in maize plants. We analyzed the transcriptome data based on Gene Ontology (GO) terms, Kyoto Encyclopedia of Genes and Genomes (KEGG) pathways, and Short Time-series Expression Miner (STEM). To verify the roles of vitamins C, E, and K in the resistance to SCMV, MCMV, or S + M infection in plants, the expression of *ZmGalDH*, *ZmAPX1*, *ZmTAT*, and *ZmNQO1* was silenced for functional validation via cucumber mosaic virus (CMV)-based virus-induced gene silencing (VIGS) assays, respectively. Subsequently, we investigated the correlation between the expression of the above four genes and the resistance to viral infections in ten selected maize inbred lines. By spraying AsA, α-tocopherol, or vitamin K1, we further determined the effects of the vitamins C, E, and K on viral infections in four maize inbred lines. These results contribute to understanding the roles of vitamins C, E, and K in conditioning viral infections in maize.

## 2. Results

### 2.1. Characterization of the Full-Length Maize Transcriptome

**Figure 1:**
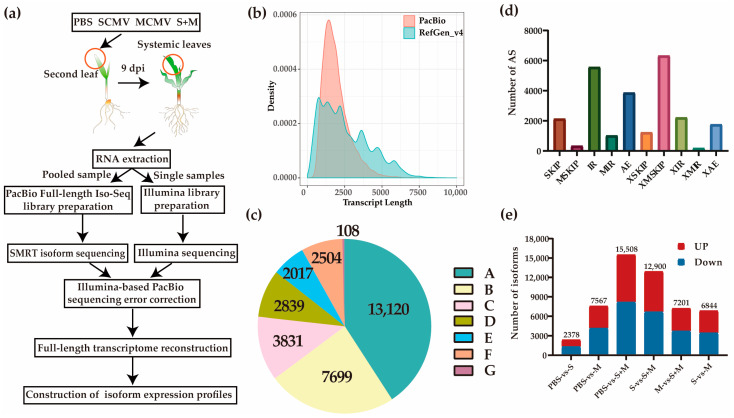
Experimental workflow and results for the full-length transcriptome. (**a**) Experimental workflow of Iso-Seq and RNA-Seq. (**b**) Comparison of transcript length between the Zm-B73-REFERENCE-GRAMENE-4.0 (RefGen_v4) and full-length transcriptome data (PacBio). (**c**) The isoforms identified based on structural comparison in Iso-Seq. A, same introns and exons; B, multiple exons and different introns; C, shorter and sequentially shared introns; D, longer and sequentially shared introns; E, lacking introns vs. containing introns, or vice versa; F, novel isoforms from novel genes; G, no overlap at the same gene loci. (**d**) Total number and categories of AS events in genes detected by Iso-Seq. (**e**) Comparison of isoform expression levels under different treatments. S, SCMV; M, MCMV.

To obtain high-confidence, full-length reference and/or specific transcripts in maize, full-length transcriptome sequencing with PacBio SMRT technology was performed using pooled samples from PBS-, SCMV-, MCMV-, and S + M-inoculated maize plants at 9 dpi (Figure 1a). RNA-Seq was used to correct the FLNC reads identified by the full-length transcriptome (Table S1). In total, 42,182 annotated loci and 134,176 annotated isoforms, as well as 18,118 new loci and 32,118 new isoforms, were identified (Figure 1b, Table S2). The total length of isoforms with multiple exons was 220,020 bp, and the total multiple-exon FLNC length was 114,677 bp (Table S3). The identified isoforms were compared with the reference genome and classified into 7 groups based on a structural comparison (Figure 1c). Among them, 40.85% (13,120) of the isoforms matched the original annotated isoform structure. There were 7699 (23.98%) “Multiple-exons and different introns” with the annotated genes/isoforms (Figure 1c). Based on the full-length transcriptome dataset, 18,960 alternative splicing (AS) events were identified in 44,030 genes, among which intron retentions (IRs) were the most abundant, accounting for 29.37% of the total AS events, followed by alternative exon ends (AEs) (20.4%), skipped exons (SKIPs) (11.27%), approximate IRs (XIRs) (11.7%), approximate AEs (XAEs) (9.3%), and approximate MIRs (XMIRs) (0.99%) (Figure 1d).

### 2.2. Differentially Expressed Isoforms (DEIs)

To explore the effects of single or co-infection of SCMV and MCMV on the expression profiles of maize transcript isoforms, we collected systemically infected leaf samples from PBS-, SCMV-, MCMV-, and S + M-inoculated maize plants at 9 dpi for RNA-Seq. According to the results, a total of 15,508 DEIs were identified in S + M libraries, followed by 7567 DEIs in MCMV libraries and 2378 DEIs in SCMV libraries, compared with PBS libraries. There were 12,900 DEIs and 7201 DEIs identified in S + M vs. SCMV and S + M vs. MCMV, respectively (Figure 1e). Moreover, 812 upregulated DEIs and 124 downregulated DEIs coexisted in SCMV, MCMV, and S + M libraries (Figure S1A,B).

To further investigate the mechanism of the maize response to SCMV, MCMV, and S + M infection, we performed GO and KEGG pathway enrichment analyses of DEIs with the same differential expression tendency. A total of 598 upregulated DEIs were obtained and enriched in ‘the response to stimulus’, ‘catalytic activity’, and ‘membrane part’ of GO terms (Figure S1C, Table S4), while 145 downregulated DEIs were identified, which were annotated with GO terms under the ‘metabolic process’, ‘catalytic activity’, and ‘organelle’ categories (Figure S1D, Table S4) The KEGG pathway analysis results revealed that the upregulated DEIs were primarily involved in ‘protein processing in the endoplasmic reticulum (ER)’, ‘MAPK signaling pathway—plant’, and ‘endocytosis’ (Figure S1E, Table S5), while the downregulated DEIs were primarily associated with ‘valine, leucine and isoleucine degradation’, ‘carbon fixation in photosynthetic organisms’, and ‘biosynthesis of secondary metabolites’ (Figure S1F, Table S5). These results indicated that both SCMV and MCMV infection could strongly trigger the host defense response against viral infections and aggravate MLN by reducing plant photosynthesis and amino acid utilization.

### 2.3. Short Time-Series Expression Miner (STEM) and Kyoto Encyclopedia of Genes and Genomes (KEGG) Pathway Enrichment Analysis

**Figure 2:**
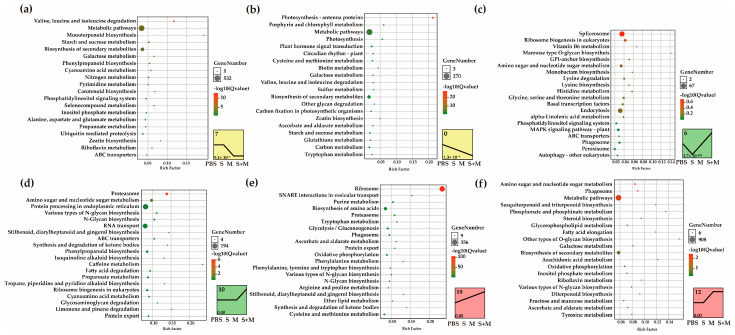
Short-Time Time-series Expression Miner (STEM) and KEGG enrichment analyses of DEIs. (**a**) The KEEG analysis of DEIs in cluster 7 of STEM analysis. (**b**) The KEEG analysis of DEIs in cluster 0 of STEM analysis. (**c**) The KEEG analysis of DEIs in cluster 6 of STEM analysis. (**d**) The KEEG analysis of DEIs in cluster 10 of STEM analysis. (**e**) The KEEG analysis of DEIs in cluster 19 of STEM analysis. (**f**) The KEEG analysis of DEIs in cluster 12 of STEM analysis. The STEM analysis chart is located at the bottom left of the figures. S, SCMV; M, MCMV.

To identify the potential regulatory mechanism responsible for gene expression changes in response to SCMV and MCMV infection, we further analyzed the correlation of gene expression among different treatments using STEM. A total of 45,767 isoforms were classified into six statistically significant trend clusters and their corresponding KEGG categories were also analyzed (Figure 2 and Figure S2, and Table S6).

There were 7047 isoforms assigned to cluster 7 and cluster 0, in which the expression trends of isoforms were either downregulated or unchanged in SCMV libraries, whereas all were downregulated in MCMV and S + M libraries. KEGG enrichment analysis revealed that the isoforms in cluster 7 were primarily enriched in ‘valine, leucine and isoleucine degradation’, ‘phenylpropanoid biosynthesis’, and ‘cyanoamino acid metabolism’, while the isoforms in cluster 0 were primarily associated with ‘photosynthesis-antenna proteins’, ‘porphyrin and chlorophyll metabolism’, and ‘plant hormone signal transduction’ (Figure 2a,b).

In addition, 3810 isoforms were assigned to cluster 6. In this cluster, the expression trend of the isoforms was downregulated in SCMV-infected maize plants but upregulated in MCMV- and S + M-infected maize plants. KEGG enrichment analysis showed that the ‘spliceosome’ pathway was significantly enriched (Figure 2c).

There were 10,085 isoforms assigned to cluster 10. In SCMV- and MCMV-infected plants, the expression trends of the isoforms were unchanged, while they were upregulated in S + M-infected plants. The isoforms related to ‘proteasomes’, ‘amino sugars’, and ‘nucleotide sugar metabolism’ were significantly enriched according to KEGG enrichment analysis (Figure 2d).

A total of 17,273 isoforms were assigned to cluster 19 and cluster 12. In SCMV libraries, the expression trend of isoforms was unchanged or upregulated, while it was upregulated in the MCMV and S + M libraries. Pathways involving ‘ribosomes’, ‘SNARE interactions in vesicular transport’, ‘proteasomes’, ‘ascorbate and aldarate metabolism’, ‘ubiquinone and other terpenoid-quinone biosynthesis’, and ‘phenylalanine metabolism’ were enriched in cluster 19 (Figure 2e). These isoforms in cluster 12 were enriched in ‘amino sugar and nucleotide sugar metabolism’, ‘phagosome’, and ‘metabolic pathways’ (Figure 2f).

To verify the accuracy of the transcriptome data, we selected five ROS-related genes and three 26 proteasome-related genes for verification through RT–qPCR. The expression levels of *ZmACC*, *ZmALDH*, *ZmCAT1*, *ZmPSMA1*, and *ZmPSMA5-T002* were upregulated in SCMV- and MCMV-infected plants and were higher in S + M-infected plants. In addition, the expression levels of *ZmMDH*, *ZmPAO1*, and *ZmPI31* were also induced after viral infections (Figure S3). These results were inconsistent with that in transcriptome data.

These results showed that single and synergistic infection induced the expression levels of antiviral-related genes that were primarily involved in ROS homeostasis, proteasome, and phenylalanine metabolism. Similar to the findings reported in other viral infections, chloroplast and biosynthesis pathways in maize were disturbed after infection with SCMV and MCMV, and more serious after co-infection.

### 2.4. Analyses of DEIs in the 26S Proteasome

Ubiquitin-mediated protein degradation by the 26S proteasome participates in plant antiviral defense or pathogenic mechanisms [42,43]. In this study, 34 DEIs involved in the 26S proteasome pathway were identified (Figure S4A,B). Thirty-three DEIs involved in the 19S regulatory particle (PA700) and 20S core particle were upregulated in single and synergistic infection, while only the regulatory particle non-ATPase subunit (*Rpn5*) was downregulated. Interestingly, the expression level of proteasome inhibitor subunit 1 (*ZmPI31*), a key gene that inhibits the maturation of the 20S proteasome to the 26S proteasome, was upregulated in single infection and co-infection, and the results of RT–qPCR were consistent with the transcriptome (Figure S3). These findings indicated that SCMV and MCMV single and synergistic infection promoted ubiquitin-mediated protein degradation by the 26S proteasome, thereby the promotion effect of S + M infection was especially strong.

### 2.5. Analyses of DEIs Involved in Photosynthesis

A virus replication complex (VRC) is an assembly of viral proteins and viral genomic RNA with essential host factors, which preferentially accumulate in the membranous structures of organelles, such as chloroplast, to disturb photosynthesis [44,45]. In this study, 108 DEIs involved in photosynthesis were identified in the S + M library, which was more than those in the SCMV (34 DEIs) and MCMV (46 DEIs) libraries (Figure S4C–G). The results showed that 9 DEIs in the cytochrome b6/f complex, 16 DEIs in F-type-ATPase, 26 DEIs in photosystem I, 30 DEIs in photosystem II, and 17 DEIs in photosynthetic electron transport pathways were significantly downregulated, while one isoform of *ZmPsbQ*, two isoforms of ferredoxin (*ZmFd*), and two isoforms of ferredoxin NADP^+^ oxidoreductase (*ZmFNR*) were significantly upregulated after S + M infection (Figure S4F,G). These findings indicated that co-infection of SCMV and MCMV caused more serious damage to plant photosynthesis than either single infection.

### 2.6. Roles of Vitamin C in Combating Viral Infection

**Figure 3:**
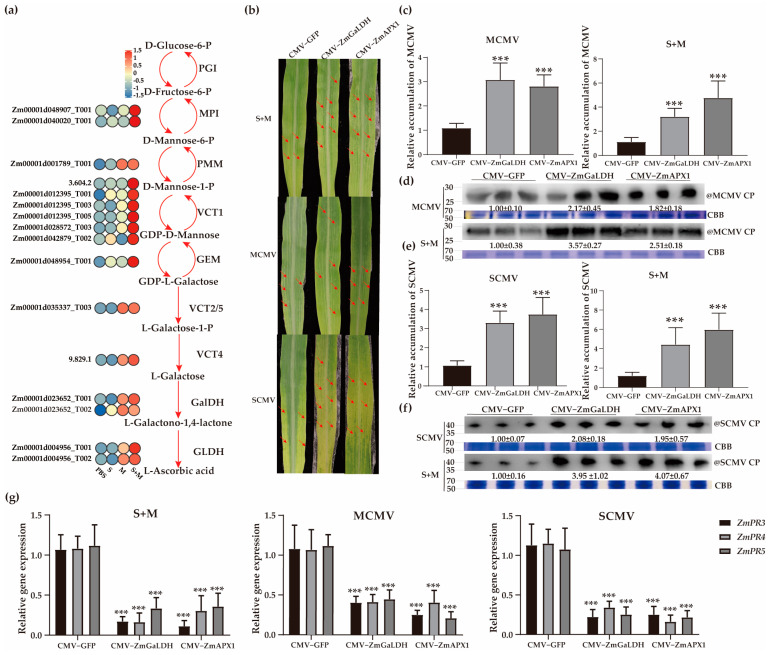
Effects of vitamin C biosynthesis-related isoforms on SCMV, MCMV, or S + M infection in maize plants. (**a**) Diagram showing the crosstalk of vitamin C biosynthesis pathway and the expression levels of corresponding genes. S, SCMV; M, MCMV. (**b**) The symptoms of the first systemically infected leaves in gene-silenced maize plants infected by S + M, MCMV, or SCMV, at 7 dpi. The red arrows indicate the mosaic or necrotic regions on systemically infected leaves. (**c**) The accumulations of MCMV RNA in gene-silenced maize plants after single and synergistic infection at 7 dpi by RT–qPCR. (**d**) The accumulations of MCMV CP proteins in gene-silenced maize plants after single and synergistic infection at 7 dpi by Western blot analyses. (**e**) The accumulations of SCMV RNA in gene-silenced maize plants after single and synergistic infection at 7 dpi by RT–qPCR. (**f**) The accumulations of SCMV CP proteins in gene-silenced maize plants after single and synergistic infection at 7 dpi by Western blot analyses. (**g**) Analyses of SA-responsive *PR* gene expression in gene-silenced maize plants after viral infections. The abbreviations of genes and compounds are listed in Table S7. CP, coat protein; CBB, Coomassie brilliant blue. Significance levels were set at *** *p* < 0.001.

The content of vitamin C usually increases under stress conditions to protect against oxidative stress through the detoxification of ROS [46]. In this study, we found that the ‘ascorbate and aldarate metabolism’ pathway was significantly enriched after viral infections (Figure 2e). Additionally, an expression pattern diagram of isoforms involved in vitamin C metabolism was constructed (Figure 3a). Among them, 16, 12, and 4 DEIs were upregulated after S + M, MCMV, and SCMV infection, respectively. To further investigate the roles of vitamin C pathway-related DEIs in the development of MLN symptoms, we chose *ZmGalDH* (Zm00001d023652_T002) and *ZmAPX1* (Zm00001d028709_T001), which were all upregulated after SCMV, MCMV, or S + M infection (Figure 3a and Figure S5), for functional verification through CMV-based VIGS assays (Figure 3b). At 7 dpi, differential symptoms appeared on the first systemically infected leaves, and *ZmGalDH*- and *ZmAPX1*-silenced plant leaves infected by SCMV, MCMV, and S + M showed more expanded mosaic and chlorosis areas than those subjected to the control treatment, respectively (Figure 3b). The RT–qPCR results showed that the silencing efficiencies of *ZmGalDH* and *ZmAPX1* were 67~76%, (Figure S6). The results indicated that silencing *ZmGalDH* or *ZmAPX1* significantly increased the accumulations of MCMV and SCMV RNA and CP proteins both in single and synergistic infected maize plants (Figure 3c–f).

Salicylic acid (SA) treatment can significantly inhibit MCMV accumulation and plays an important role in preventing MCMV infection in maize [47]. To investigate whether *ZmGaLDH* or *ZmAPX1* affected the expression of SA-related *PR* genes after viral infections, we analyzed the expression levels of *ZmPR3*, *ZmPR4*, and *ZmPR5* in the gene-silenced maize plants described above. The results showed that the accumulations of *ZmPR3*, *ZmPR4*, and *ZmPR5* were significantly reduced in *ZmGalDH*- or *ZmAPX1*-silenced plants (Figure 3g). These results suggested that SA levels were decreased in *ZmGalDH*- or *ZmAPX1*-silenced plants, which enhanced the sensitivity of maize to SCMV, MCMV, and S + M infection.

### 2.7. Roles of Vitamins E and K in Antiviral Responses

**Figure 4:**
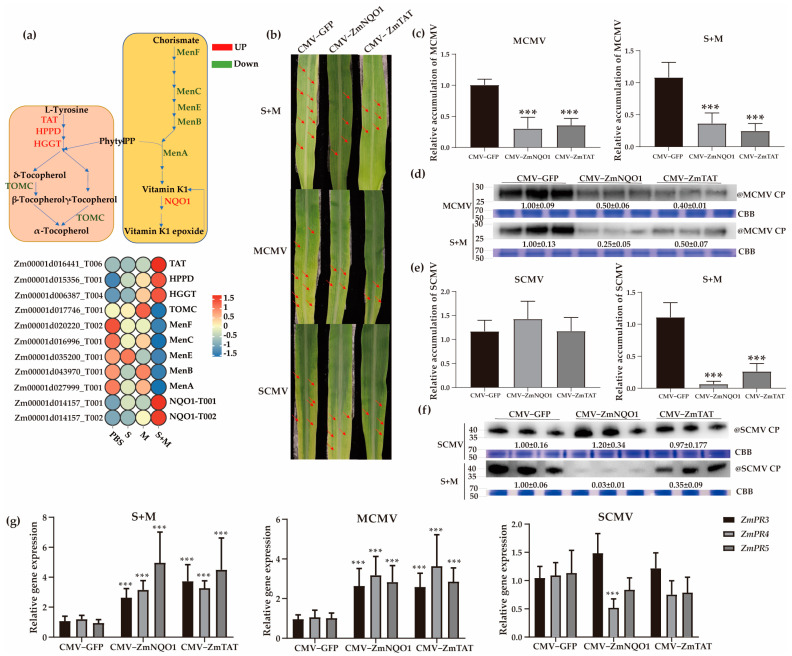
Effects of vitamins E and K biosynthesis-related genes on SCMV, MCMV, or S + M infection in maize plants (**a**) Diagram showing the crosstalk of vitamins E and K biosynthesis pathway and the expression levels of corresponding genes. (**b**) The symptoms of the first systemically infected leaves in gene-silenced maize plants infected by SCMV, MCMV, or S + M at 7 dpi. The red arrows indicate the mosaic or necrotic regions on systemically infected leaves. (**c**) The accumulations of MCMV RNA in gene-silenced maize plants after single and synergistic infection at 7 dpi by RT–qPCR. (**d**) The accumulations of MCMV CP proteins in gene-silenced maize plants after single and synergistic infection at 7 dpi by Western blot analyses. (**e**) The accumulations of SCMV RNA in gene-silenced maize plants after single and synergistic infection at 7 dpi by RT–qPCR. (**f**) The accumulations of SCMV CP proteins in gene-silenced maize plants after single and synergistic infection at 7 dpi by Western blot analyses. (**g**) Analyses of SA-responsive *PR* gene expression in gene-silenced maize plants after viral infections. The abbreviations of genes and compounds are listed in Table S7. CP, coat protein; CBB, Coomassie brilliant blue. Significance levels were set at *** *p* < 0.001.

It has been reported that several vitamins, including vitamin E, can induce disease resistance in plants [48]. In this study, we found that the ‘ubiquinone and other terpenoid-quinone biosynthesis’ pathway was significantly enriched (Figure 2e). The expression patterns of vitamin E- and K-related genes were determined during viral infections. The results demonstrated that the expression of *ZmTAT*, *ZmHPPD*, and *ZmHGGT*, three upstream genes related to vitamin E biosynthesis, were significantly upregulated, while the downstream gene *ZmTOMC* was significantly downregulated in SCMV-, MCMV-, and S + M-infected maize plants, especially in S + M (Figure 4a). In contrast, the upstream genes involved in vitamin K biosynthesis, *ZmMenA*, *ZmMenB*, *ZmMenC*, *ZmMenE*, and *ZmMenF*, were significantly reduced, while *ZmNQO1* was significantly induced after viral infections (Figure 4a).

To determine the antiviral roles of the genes related to vitamins E and K, CMV-VIGS assays were performed to transiently silence *ZmNQO1* or *ZmTAT*. At 7 dpi, *ZmNQO1* or *ZmTAT*-silenced plants infected by MCMV or S + M exhibited fewer expanded chlorosis areas compared with the control plants. However, the symptoms of the first systematic leaves did not change significantly in *ZmNQO1*- or *ZmTAT*-silenced plants after SCMV infection (Figure 4b). The silencing efficiencies of *ZmNQO1* and *ZmTAT* in silenced plants were 55%~99% (Figure S7). The results showed that the accumulations of MCMV RNA and CP proteins were significantly reduced both in *ZmNQO1*- and *ZmTAT*-silenced plants infected by MCMV or S + M (Figure 4c,d). The accumulations of SCMV RNA and CP proteins were not changed after SCMV infection, while they were significantly reduced after S + M infection both in *ZmNQO1*- and *ZmTAT*-silenced plants through RT–qPCR and Western blot assays (Figure 4e,f). Moreover, the results showed that silencing *ZmNQO1* and *ZmTAT* significantly increased the expression levels of *ZmPR3*, *ZmPR4,* and *ZmPR5* in MCMV- and S + M-infected maize plants, respectively (Figure 4g). However, silencing *ZmNQO1* or *ZmTAT* did not affect the expression levels of *ZmPR3*, *ZmPR4*, and *ZmPR5* in SCMV-infected plants, except for *ZmPR4* in *ZmNQO1*-silenced plants (Figure 4g).

### 2.8. Identification of MLN-Resistant Maize Inbred Lines

The use of virus-resistant hybrids and cultivars is the most environmentally sustainable and economically viable approach for controlling viral diseases in crops [49]. In 2020 and 2021, the resistance MCMV and SCMV resistance of 54 maize inbred lines at the seedling stage were tested twice in each experiment. The results showed that the relative disease index ranged from 15.74% to 94.91% in MCMV-infected maize plants, of which S766 was the only resistant inbred line. Two inbred lines, H21 and M14, showed moderate resistance, and the symptoms after systemic infection were alleviated in high temperatures (≥40 °C). Surprisingly, the inbred lines Ji853, Huangye4, and S951 showed lethal necrosis on leaves at 15 dpi, which was similar to the MLN symptom (Table S8, Figure S8). As expected, SCMV-resistant inbred lines were widely found. The relative disease index of 54 maize inbred lines by SCMV ranged from 0.79% to 74.70%. Among them, three (5.56%) inbred lines showed high resistance and five (9.26%) were resistant (Table S8, Figure S9).

**Figure 5:**
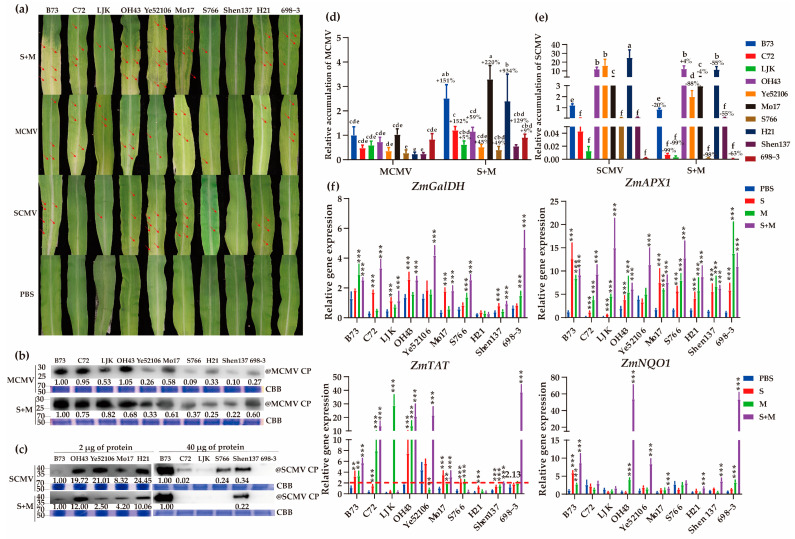
Identification of MLN-resistant maize inbred lines and correlation between vitamin-related gene expressions and viral infections. (**a**) The symptoms of the first systemically infected leaves of ten inbred lines inoculated with PBS, SCMV, MCMV, or S + M at 9 dpi. The red arrows indicate the mosaic or necrotic regions on systemically infected leaves. (**b**) The accumulations of MCMV CP proteins in MCMV- or S + M-infected maize leaves at 9 dpi by Western blot analyses. CP, coat protein; CBB, Coomassie brilliant blue. (**c**) The accumulations of SCMV CP proteins in SCMV- or S + M-infected maize leaves at 9 dpi by Western blot analyses. (**d**) The accumulations of MCMV RNA in MCMV- or S + M-infected maize leaves at 9 dpi by RT–qPCR. The numbers of ‘±’ symbols indicate the upregulated or downregulated percentage of viral RNAs. Lowercase letters indicate significant difference (*p* < 0.05). (**e**) The accumulations of SCMV RNA in SCMV- or S + M-infected maize leaves at 9 dpi by RT–qPCR. The numbers of ‘±’ symbols indicate the upregulated or downregulated percentage of viral RNAs. Lowercase letters indicate significant difference (*p* < 0.05). (**f**) The accumulations of different vitamin-related genes in ten inbred lines inoculated with PBS, SCMV, MCMV or S + M at 9 dpi by RT–qPCR. The lower level of relative gene expression of *ZmTAT* was marked with a red dashed line (2.13). Significance levels were set at ** *p* < 0.01; and *** *p* < 0.001.

To further verify the correlations between the single and synergistic viral infection results, ten (relatively highly resistant to MCMV in 54) inbred lines were selected and inoculated with SCMV, MCMV, and S + M. The results showed that the first systemically infected leaves of all maize plants except for those of S766 and Shen137 showed severe mosaic and chlorosis after MCMV and S + M infection (Figure 5a). After SCMV infection, B73, OH43, Ye5216, MO17, and H21 exhibited more expanded chlorosis areas than other inbred lines (Figure 5a). The symptoms caused by co-infection were more severe than those caused by a single infection with SCMV or MCMV (Figure 5a).

Among the MCMV-infected plants, the accumulations of MCMV RNA and CP proteins were significantly decreased in S766, H21, and Shen13 compared with that in B73 according to RT–qPCR and Western blotting (Figure 5b,d). Moreover, the results indicated that C72, LJK, S766, Shen137, and 698-3 accumulated lower SCMV titers than B73, while more SCMV titers accumulated in OH43, Ye5216, Mo17, and H21 inbred lines (Figure 5c,e). In S + M-infected plants, the levels of MCMV RNA in all inbred lines were higher than that in MCMV-infected plants (Figure 5d). Among them, the accumulation of MCMV RNA in S + M-infected H21 plants was significantly increased by 9-fold compared with that in MCMV-infected plants. The accumulation of SCMV RNA in co-infected plants was lower than that in SCMV-infected plants, except for OH43 and Mo17 (Figure 5e).

We subsequently investigated the relationships between the crucial vitamin-related genes and virus resistance in these 10 inbred lines (Figure 5f). The expression level of *ZmGaLDH* was significantly upregulated in S + M-infected plants, except for H21 (Figure 5f). The accumulation of *ZmAPX1* was significantly upregulated after SCMV and MCMV single and synergistic infection in 9 inbred lines, except for Ye52106 (Figure 5f). The expression level of *ZmTAT* was relatively low in SCMV-resistant inbred lines C72, LJK, S766, Shen137, and 698-3 compared to B73. Additionally, the low expression of *ZmTAT* was also determined in MCMV-resistant inbred lines, including S766, H21, and Shen137, infected with MCMV and S + M-resistant inbred lines, and S766 and Shen137, infected with S + M (Figure 5f). However, *ZmNQO1* expression was induced in only a few inbred lines after MCMV or S + M infection (Figure 5f). These findings indicated that ZmAPX1 is widely involved in response to viral infections, while *ZmGalDH* is more sensitive to co-infection than single infection. Interestingly, the expression of *ZmTAT* was negatively correlated with the accumulation of SCMV and MCMV RNA in resistant inbred lines.

### 2.9. Effects of Spraying Different Vitamins on Viral Infections in Resistant and Susceptible Maize Inbred Lines

Exogenous spraying with vitamins can improve plant resistance to biotic and abiotic stresses [50]. To determine the effects of exogenous vitamin treatments on viral infections in maize plants, certain concentrations of vitamin C, E or K were sprayed on the inbred lines B73 and OH43 (susceptible), and S766 and Shen137 (resistant).

**Figure 6:**
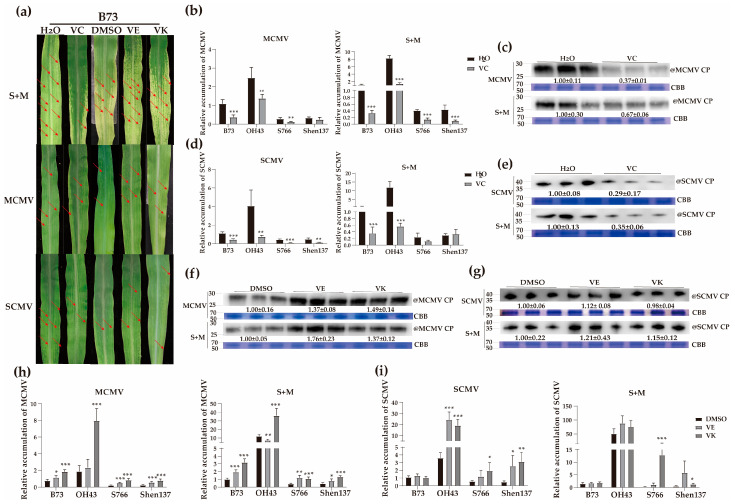
Effects of spraying different vitamin solutions on virus infections in resistant and susceptible maize inbred lines. (**a**) First systemically infected leaves of B73 plants inoculated with SCMV, MCMV, or S + M at 9 dpi. The red arrows indicate the mosaic or necrotic regions on systemically infected leaves. (**b**) The accumulations of MCMV RNA in MCMV- or S + M-infected maize leaves at 9 dpi after spraying vitamin C by RT–qPCR. (**c**) The accumulations of MCMV CP proteins in MCMV- or S + M-infected B73 maize leaves at 9 dpi after spraying vitamin C by Western blot analyses. (**d**) The accumulations of SCMV RNA in SCMV- or S + M-infected maize leaves at 9 dpi after spraying vitamin C by RT–qPCR. (**e**) The accumulations of SCMV CP proteins in SCMV- or S + M-infected B73 maize leaves at 9 dpi after spraying vitamin C by Western blot analyses. (**f**) The accumulations of MCMV CP proteins in MCMV- or S + M-infected B73 maize leaves at 9 dpi after spraying vitamin E or K by Western blot analyses. (**g**) The accumulations of SCMV CP proteins in SCMV- or S + M-infected B73 maize leaves at 9 dpi after spraying vitamin E or K by Western blot analyses. (**h**) The accumulations of SCMV RNA in MCMV- or S + M-infected maize leaves at 9 dpi after spraying vitamin E or K by RT–qPCR. (**i**) The accumulations of SCMV RNA in SCMV- or S + M-infected maize leaves at 9 dpi after spraying vitamin E or K by Western blot analyses. CP, coat protein; CBB, Coomassie brilliant blue; VC, vitamin C; VE, vitamin E; VK, vitamin K1. Significance levels were set at * *p* < 0.05; ** *p* < 0.01; and *** *p* < 0.001.

At 7 dpi, B73 plant leaves treated with vitamin C showed fewer expanded mosaic and chlorosis areas after SCMV, MCMV, and S + M infection, respectively (Figure 6a). RT–qPCR results indicated that the accumulations of MCMV RNA were reduced after spraying vitamin C in both resistant and susceptible inbred lines infected with MCMV or S + M (except for Shen137 infected by MCMV) (Figure 6b). Moreover, the relative expression levels of SCMV RNA were also significantly reduced after spraying vitamin C, except for S766 and Shen137 infected by S + M (Figure 6d). The results of Western blotting showed that the accumulations of MCMV and SCMV CP were consistently reduced after spraying vitamin C on virus-infected B73 plants (Figure 6c,e). In MCMV- and S + M-infected plants, both vitamin E and K spraying caused more severe chlorosis on the first systemically infected leaves, respectively (Figure 6a). However, SCMV-infected plants showed no obvious changes in symptoms after spraying vitamin E or K (Figure 6a). In MCMV- and S + M-infected plants, the accumulations of MCMV RNA were significantly increased in both resistant and susceptible inbred lines after spraying vitamin E or K, except for the susceptible inbred line OH43 treated with vitamin E (Figure 6h), and the protein levels of MCMV CP also increased significantly in B73 plants sprayed with vitamin E or K (Figure 6f). The accumulations of SCMV RNA were induced in OH43 and Shen137 infected with SCMV and S + M after spraying vitamin E or K, and S766 infected with SCMV and S + M after spraying vitamin K, while SCMV CP protein levels remained unchanged in B73 plants infected with SCMV or S + M after spraying vitamin E or K (Figure 6g,i). Moreover, we measured the expression levels of SA-related *PR* genes in different treatments, and the results revealed that the expression levels of *ZmPR3*, *ZmPR4,* and *ZmPR5* were significantly increased in SCMV-, MCMV-, and S + M-infected B73 plants after vitamin C treatment (Figure S10), while decreased after spraying vitamin E or K (Figure S11). These results indicated that vitamin C improved resistance to viral infections positively by regulating the expression of SA-related *PR* genes, while vitamins E and K had opposite effects.

## 3. Discussion

In many regions of the world, maize production is severely affected by MLN, especially in Africa [51]. SCMV, the predominant maize-infecting potyvirus, is distributed worldwide, so outbreaks of MLN tend to be caused by MCMV emergence [2]. In maize, the main resistance genes, the interaction between SCMV and maize plants, and the pathogenic mechanism of SCMV have been extensively studied [3,6,7,8]. However, relatively few research studies have been implemented to explore the resistance mechanism of maize to MCMV and MLN, especially MLN [2]. In this study, we combined Iso-Seq and RNA-Seq data from maize infected with SCMV, MCMV, or S + M to find new ideas for anti-MLN (Figure 1a). The results showed that the expression levels of isoforms, which changed in maize after co-infection, were more complex than that after infection with a single virus (Figure 1e). The STEM analysis results revealed that SCMV and MCMV played different roles in co-infection, providing further evidence for their effects on MLN (Figure 2 and Figure S2). The results also demonstrated that the genes in five of the six clusters showed the same expression pattern in MCMV and S + M libraries (Figure S2), suggesting that MCMV might play a major role in the synergistic infection.

As a result of plant viral infections, photosynthesis is often disrupted. SCMV and MCMV infections have been shown to negatively regulate plant photosynthesis [6,12,52]. Similarly, we found that the synergistic infection of SCMV and MCMV caused more serious effects on photosynthesis (Figure 2b and Figure S4C–G). Specifically, the expression of a chloroplast gene, ferredoxin 3 (*ZmFD3*, Zm00001d034760_T001), was significantly upregulated after S + M infection (Figure S3). Ferredoxin 1 has been reported to interact with p25 of potato virus X (PVX) and inhibit its infection in tobacco [53]. Moreover, ZmFD5 can interact with HC-Pro of SCMV by a yeast two-hybrid method [54]. Therefore, the roles of ZmFD3 in antiviral responses require to be further investigated.

In the arms race between pathogens and hosts, viral infections cause chlorosis, mottling, dwarfism, and even death in plants [51]. In response to viral infections, plants have evolved a variety of strategies to alleviate virus-induced symptoms [55]. In this study, we found that ‘phenylalanine metabolism’, ‘the proteasome’, and ‘ascorbate and aldarate metabolism’, which have been reported to be involved in antiviral activity [56,57,58], were significantly enriched, and the expression levels of most isoforms in these pathways were upregulated after viral infections, especially S + M infection (Figure 2e). It has been reported that the knockdown of phenylalanine ammonia-lyases (*ZmPAL*) expression leads to enhanced SCMV infection symptom severity and virus multiplication [59].

Vitamins are considered important antioxidants in plants [60]. Vitamin C is the most abundant and ubiquitous cellular antioxidant that scavenges many types of ROS, such as O^2−^, ^1^O_2_, and H_2_O_2_ [61,62], and has been detected in the majority of plant cell types, organelles, and apoplasts [63]. In this study, we silenced the expression of vitamin C-related genes (*ZmGalDH* and *ZmAPX1*) using a CMV-VIGS vector and found that after SCMV-, MCMV-, or S + M-infection, the chlorosis areas and virus accumulations were increased in gene-silenced maize plants (Figure 3b–f). In addition, the results of RT–qPCR showed that the expression levels of *ZmGalDH* and *ZmAPX1* in 10 resistant inbred lines were increased after viral infections (Figure 5f). The virus accumulations in four inbred lines with different sensitivities to SCMV or MCMV were decreased after spraying vitamin C (Figure 6b–e). The findings suggested that vitamin C plays an important role in resistance to viral infections in maize. Vitamin C has been reported to function as a double-edged sword in the antiviral process of plants. On the one hand, the content of vitamin C decreased in the *TaVTC2-RNAi* mutant plant, in which the burst of ROS caused resistance to WYMV infection [26]. On the other hand, vitamin C increased the level of JA in radish, which enhanced its resistance to TuMV [31]. Therefore, the antiviral roles of vitamin C in resistance to virus infections in maize need to be further investigated.

Vitamin E affords protection to membranes primarily by quenching singlet oxygen and reacting with lipid peroxy radicals and has been shown to reduce the amount of lipid peroxidation in leaves and seeds [36,64]. Vitamin K acts in redox reactions at the plasma membrane of plants. Although vitamin K has been reported to play a role in the redox reaction of the plant plasma membrane [40], its specific role in scavenging ROS in plants needs to be further clarified. The SCMV and MCMV titers were increased significantly in *ZmTAT*- or *ZmNQO1*-silenced plants infected with MCMV or S + M (Figure 4b–f). RT–qPCR showed that the absolute value of *ZmTAT* expression remained low (<2.13) in the MCMV-resistant inbred lines (Figure 5f). The results showed that vitamin E and vitamin K could positively regulate the accumulation of MCMV in maize infected with MCMV or S + M, while it did not affect the accumulation of SCMV. It has been reported that exogenous vitamin E spraying could increase the tolerance to TMV in tomato plants [35]. In this study, the foliar application of vitamins E and K promoted MCMV infection, possibly by regulating the content of ROS that could enhance MCMV infection [47]. Surprisingly, different vitamins play different roles in the interactions between viruses and plants, while the molecular mechanisms remain unclear.

**Figure 7:**
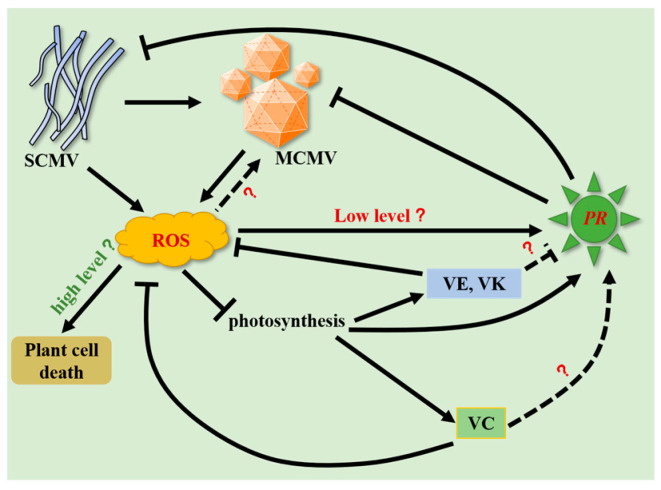
A schematic diagram depicting the possible roles of vitamins C, E, and K in regulating viral infections in maize. VC, vitamin C; VE, vitamin E; VK, vitamin K; PR, salicylic acid (SA)-related pathogenesis-related (*PR*) genes.

Spraying SA on pepper fruit has been shown to increase vitamin C content [65]. Exogenous vitamin C can be an effective antiviral agent against TuMV in *Brassica rapa* [66]. The increase in SA content in Arabidopsis *vtc1-1* mutants (which are deficient in vitamin C) was associated with increased resistance to the strong bacterial pathogen *Pseudomonas syringae*, but its accompanying high concentration of ROS made the leaves more prone to programmed death [27,67]. Compared with normal plants, SA-deficient plants presented higher levels of α- and γ-tocopherols (vitamin E) [68]. SA and a wide range of vitamins, including vitamins E and K, depend on shikimate pathway metabolites as precursors [61,69]. SA treatment significantly inhibited the accumulation of MCMV in maize [47]. Similar results were also obtained in SCMV-infected maize plants [59]. Since SA is closely related to antiviral activity and vitamins, we detected the expression of SA-related *PR* genes. Vitamin C plays a positive role in regulating SA-related *PR* genes in SCMV, MCMV, or S + M infection (Figure 3b–g, Figure 6b–e, and Figure S9). In contrast, vitamin E or K plays a negative role in regulating SA-related *PR* genes in MCMV, or S + M infection (Figure 4b–g, Figure 6f–h, and Figure S10). The mechanisms underlying the regulation of vitamins on the expression levels of *PR* genes to participate in antiviral responses in maize need to be further investigated (Figure 7).

In this study, the isoform expression profiles of maize in response to SCMV, MCMV, and S + M infection were obtained. The DEIs were primarily involved in photosynthesis, ROS scavenging, and disease resistance. Moreover, the different roles of vitamins C, E, and K in resistance to virus infections in maize were explored, which possibly function by maintaining the homeostasis of ROS and SA (Figure 7). The inbred lines Ji853, Huangye4, and S951 infected with MCMV alone could cause MLN symptoms, which provides valuable germplasm resources to investigate MCMV and MLN pathogenesis in more detail. In addition, we found the MLN-resistant inbred lines S766 and Shen137, which provided resistant germplasm resources for disease-resistant breeding. This study contributes to the elucidation of SCMV and MCMV pathogenesis and the defense responses of maize to virus infections.

## 4. Materials and Methods

### 4.1. Plant Growth and Virus Inoculation

*Nicotiana benthamiana* plants and maize (*Zea mays* L.) inbred line B73 used in CMV-based VIGS assays were grown in growth chambers (24 °C/22 °C, day/night; 16 h/8 h, light/dark). The source preparation and inoculation method of SCMV and MCMV single and synergistic infections were in accordance with our previous report [13]. First, 0.1 g of maize leaves infected with SCMV or MCMV were ground into a powder in liquid nitrogen and transferred to a 1.5 mL centrifuge tube, after which 800 μL of 1.0 mM phosphate buffer saline (PBS, pH 7.2) was added. After shaking and mixing for 5 min, the samples were centrifuged (4 °C, 5000× *g*, 5 min). Then, 500 μL of the supernatant was transferred to a new 1.5 mL tube. The inoculant for co-infection consisted of a mixture of crude extracts from SCMV- and MCMV-infected maize plants (15 μL each). At 9 days post inoculation (dpi), the systemically infected leaves were sampled and subjected to gene expression analysis.

### 4.2. SMRT Iso-Seq and RNA-Seq

An overview of the experimental procedure is illustrated in Figure 1a. In brief, the total RNA was extracted from the systemically infected maize leaves of mock (PBS), SCMV, MCMV, and S + M treatments at 9 dpi using an EastepTM Super Total RNA Extraction Kit (Promega, Shanghai, China). All the samples included in the subsequent analysis showed an RNA integrity number (RIN) exceeding 9.0 upon quantification and analysis with an Agilent Bioanalyzer 2100 (Agilent Technologies, Palo Alto, CA, USA). The RNA was pooled equally to reverse transcribe into cDNA using a SMARTer™ PCR cDNA Synthesis Kit (Clontech Laboratories, Inc., Palo Alto, CA, USA) for full-length Iso-Seq on the PacBio Sequel II platform (Frasergen, Wuhan, China). The sequence data were processed using SMRT Link 5.0 software with the following parameters: Minimum subread length = 50; minimum number of passes = 1; minimum predicted accuracy = 0.8; and minimum read score = 0.65 [70]. Accurate quantification of the resulting libraries was performed using Qubit 2.0 [71].

Second-generation transcriptomic data were used to correct the Iso-Seq consensus sequences by LoRDEC [72]. Twelve libraries were constructed from PBS, SCMV, MCMV, or S + M treatment using the TruSeq stranded Library Preparation kit (Illumina, San Diego, CA, USA) and sequenced using the Illumina HiSeq TM2000 platform (Illumina, San Diego, CA, USA) with standard protocols. Sequence data were deposited in the NCBI GenBank under the BioProject ID PRJNA921723.

### 4.3. Mapping to the Reference Genome

The corrected high-quality before-and-after consensus sequences were then mapped to the B73 reference genome (Zm-B73-REFERENCE-GRAMENE-4.0) using the Genomic Mapping and Alignment Program (GMAP) [73]. Error correction analysis allowed the accurate mapping of full-length nonchimeric (FLNC) reads to a reference genome, including the start sites, termination sites, and exon or intron splicing sites. The isoforms could be identified according to the alignment position of each FLNC read.

### 4.4. Analysis of AS Events

The ASprofile software v1.0 (http://ccb.jhu.edu/software/ASprofile/) was utilized to identify AS events [74,75]. Based on the output from the ASprofile, six major types of AS events were identified: (A) Exon skipping (SKIP) and cassette exons (MSKIP), (B) retention of single (IR) and multiple (MIR) introns, (C) alternative exon ends (5’, 3’, or both) (AE), (D) approximate exon skipping (XSKIP) and cassette exons (XMSKIP), (E) approximate retention of single (XIR) and multiple (XMIR) introns, and (F) approximate alternative exon ends (XAE).

### 4.5. Analysis of DEIs

Different duplicate copies in family genes and AS events produce different isoforms after viral infections [76], so all the analyses in this article were based on the data of isoforms. The reads produced by RNA-Seq were aligned using Bowtie 2 v2.2.2 [77]. The read count values were directly obtained and converted to fragments per kilobase of transcript per million mapped reads (FPKM) values by using RSEM (v1.3.0) [78]. Then, the DEIs were obtained with the standardization method DESeq2 using edge R [79]. The significant DEIs were screened at false discovery rates (FDRs) < 0.05 and absolute log2(fold-change) > 1.

### 4.6. Isoform Functional Annotation

The functions of the isoforms associated with the mapped reads were annotated based on the following databases: The NCBI nonredundant protein sequences (NR), EuKaryotic Orthologous Groups (KOG) (http://www.ncbi.nlm.nih.gov/COG/), GO (http://www.geneontology.org), Swiss-Prot (http://www.expasy.org/sprot/), and KEGG (http://www.genome.jp/kegg). All the websites of KOG, GO, Swiss-Prot, and KEGG were accessed on 8 July 2019. Swiss-Prot and KEGG annotations were determined with Diamond BLASTX 0.8.33 and KOBAS 3.0 software, respectively [80,81].

### 4.7. STEM Analysis

To analyze changes in isoform expression in different samples, all the expressed isoforms identified by RNA-Seq were grouped into 20 clusters according to their expression levels at different stages by STEM software v1.3.11 (http://www.sb.cs.cmu.edu/stem/) on the OmicShare platform (http://www.omicshare.com/tools). The details for STEM analysis refer to a previous report [82]. The isoform expressions in SCMV, MCMV, and S + M libraries were normalized according to their log2 (fold-change) values in comparison to those in PBS libraries. The differential expression analysis was performed using a hypergeometric distribution as implemented by DESeq2 with cutoffs of *p* < 0.05 and absolute log2 (fold-change) > 1.

### 4.8. CMV-Based VIGS Assays

CMV-VIGS in maize was conducted as described elsewhere [83]. The first true leaves of the gene-silenced plant were challenged by inoculation with SCMV, MCMV, and S + M (16 h/24 °C, light; 8 h/20 °C, dark). The first systemically infected leaf from each treated plant was harvested at 7 dpi to determine gene silencing efficiency and virus accumulation.

### 4.9. Evaluation of Maize Germplasm for Resistance to SCMV, MCMV, and MLN in Maize

In 2020 and 2021, 54 maize inbred lines that are commonly used in the breeding of maize in China were selected, and the seeds were preserved by Jiangsu Academy of Agricultural Sciences (Tables S8 and S9). Inoculations of SCMV and MCMV were conducted 10 days after planting in a glasshouse of Shenyang Agricultural University (room temperature). The disease classification method of MCMV was conducted based on the Maize and Wheat Improvement Centre (CIMMYT) MLN scale [84], and the classification method of SCMV disease was performed as described previously [85]. Observations and identification were performed from 6 dpi to 15 dpi, and independent experiments were performed twice.

Ten inbred lines that were relatively resistant to MCMV but not highly resistant to SCMV were selected from 54 inbred lines for MLN resistance testing. Inoculation experiments with SCMV, MCMV, and S + M were performed in an artificial climate chamber (25 °C; 16 h/8 h, light/dark). After 9 dpi, each fully expanded second systemically infected leaf was sampled and subjected to gene expression analysis.

### 4.10. Chemical Treatments

Two-leaf-stage maize plants were sprayed with 1 mM AsA (Sigma–Aldrich, St. Louis, MO, USA) diluted with ddH_2_O, or 1 mM α-tocopherol (MCE, Bloomfield, NJ, USA) or 2 mM vitamin K1 (MCE, Bloomfield, NJ, USA) diluted with DMSO containing 0.2% (*v*/*v*) Tween-20 at 24 h before and after SCMV and/or MCMV inoculation. The first systemically infected maize leaves were harvested at 7 dpi and used to analyze viral accumulation and gene expression. Three independent experiments were performed.

### 4.11. Reverse Transcription--Quantitative PCR (RT–qPCR) Analysis

Total RNA was extracted from maize leaves using an EastepTM Super Total RNA Extraction Kit (Promega, Shanghai, China). First-strand cDNA was synthesized using 1 μg of total RNA, oligo (dT18) primer and random primers, and M-MLV reverse transcriptase (Vazyme, Nanjing, China) in each 20 μL reaction. RT–qPCR was performed using the ChamQ Universal SYBR RT–qPCR Master Mix (Vazyme, Nanjing, China) on an ABI 7500 FAST Real-Time System (Applied Biosystems, Foster City, CA, USA). The specific primers (Table S10) were designed using Primer 5 software [86]. The expression of the *ZmUBI* (XM_008647047) gene served as an internal control, and relative gene expression levels were calculated using the 2^−ΔΔCt^ method.

### 4.12. Western Blotting

For Western blot assays, the total protein was extracted from the treated samples using a Plant Protein Extraction Kit (Beyotime Biotechnology, Shanghai, China) and quantified using the bicinchoninic acid (BCA) Protein Assay Kit (Beyotime Biotechnology, Shanghai, China). The total protein was separated by 12% SDS–PAGE electrophoresis and transferred to 0.2 mm polyvinylidene fluoride (PVDF) membranes (Millipore, Billerica, MA, USA), and detected by immunoblotting with SCMV or MCMV monoclonal antibodies (LV BAO, Chengdu, China), followed by an anti-mouse IgG horseradish peroxidase (HRP) antibody (ABclonal, Wuhan, China). The membranes were transferred to a solution of the chemiluminescent substrate CDP-star (Roche, Grenzach-Wyhlen, Germany), and the resulting signals were detected on a 5200 chemical luminous imaging system (Tanon, Shanghai, China).

### 4.13. Statistical Analysis

The least significant difference method (LSD) and Q test method in SPSS Version 20.0 (SPSS software, Chicago, IL, USA) were applied to analyze the significance of the differences in the RT–qPCR data. Duncan’s new multiple range method (DMRT) was used to analyze the significance of the control effect in the field test. The results are expressed as the mean ± SD.

## Data Availability

The datasets presented in this study can be found in online repositories. The names of the repository/repositories and accession number(s) can be found below: https://www.ncbi.nlm.nih.gov/, PRJNA921723. MCMV isolate KS1 (NC_003627), SCMV isolate Beijing (AY042184), *ZmGalDH* (Zm00001d023652_T002), *ZmAPX1* (Zm00001d028709_T001), *ZmTAT* (Zm00001d016441_T006), *ZmNQO1*(Zm00001d042061_T001).

## Supplementary Materials

The following supporting information can be downloaded at: https://www.mdpi.com/article/10.3390/ijms24098012/s1.
