# Supplementary material for: Transcriptomic and Functional Analyses Reveal the Different Roles of Vitamins C, E, and K in Regulating Viral Infections in Maize
Source: Int J Mol Sci. 2023 Apr 28;24(9):8012. doi: 10.3390/ijms24098012 (PMC10178231; doi:10.3390/ijms24098012)
Supplement: Supplementary file 1 [file ijms-24-08012-s001.zip › ijms-2343195-supplementary/Figure S1-S11.pdf]

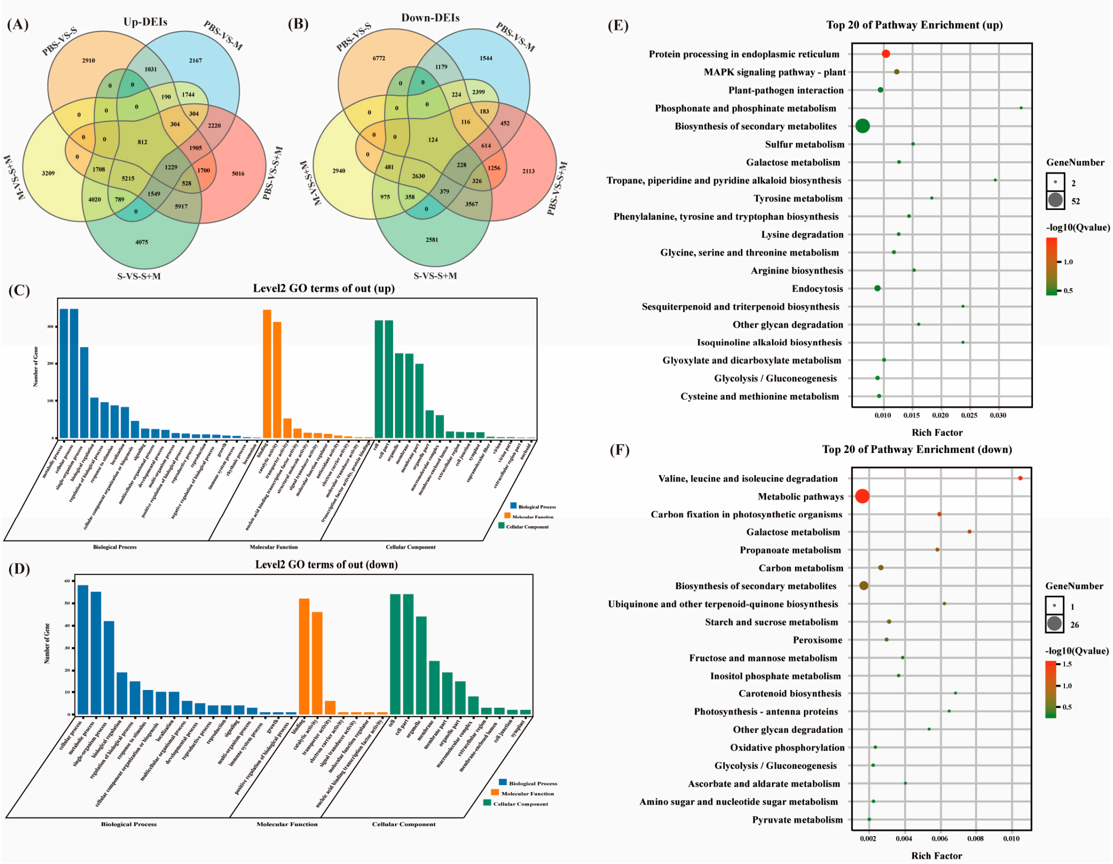

Figure S1: Statistical and enrichment analyses of DEIs. (A) Venn diagrams showing total up-regulated DEIs and DEIs in five different comparison groups. (B) Venn diagrams showing total down - regulated DEIs and DEIs in five different comparison groups. (C) Results of GO analysis of isoforms that were up- regulated under different treatments. (D) Results of GO analysis of isoforms that were down-regulated under different treatments. (E) Results of KEGG analysis of isoforms that were up-regulated under different treatments. (F) Results of KEGG analysis of isoforms that were down-regulated under different treatments.

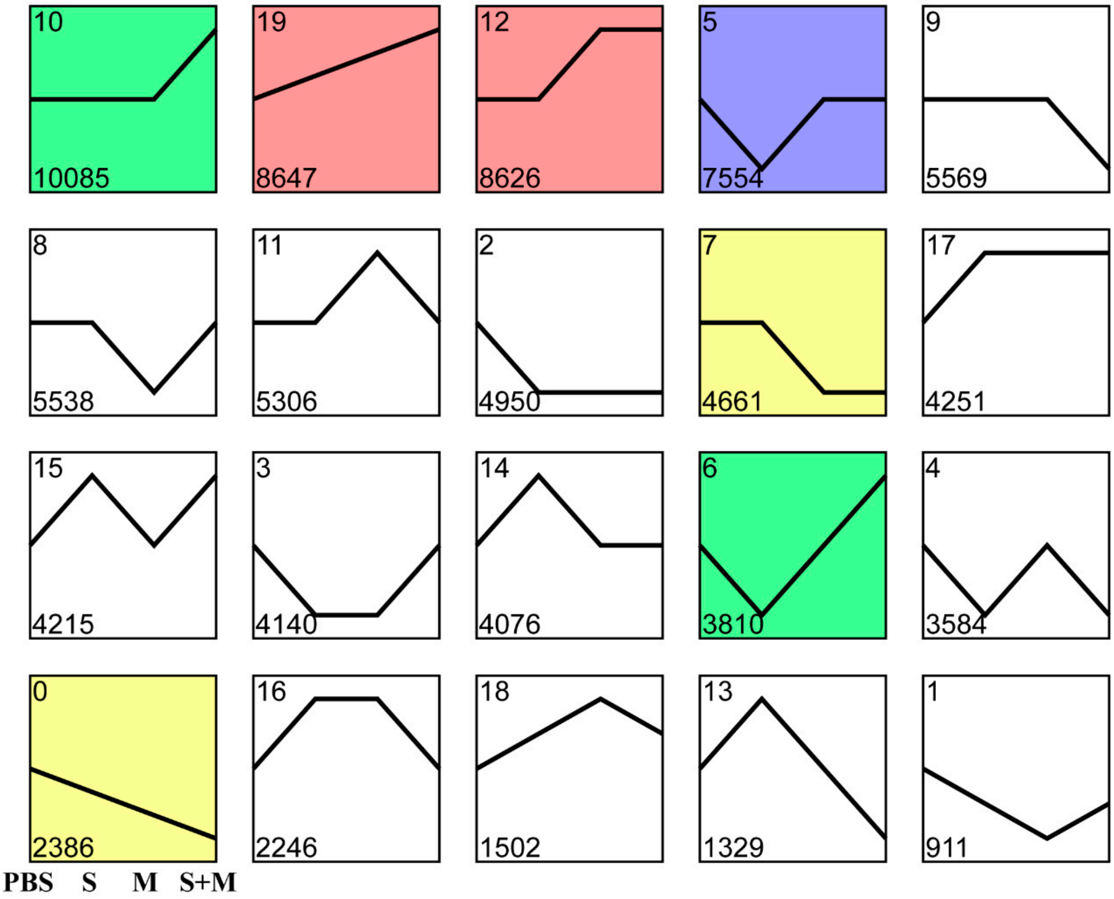

Figure S2: The clusters according to their expression profiles. All of the expressed isoforms identified by RNA-Seq were clustered into 20 clusters according to their expression profiles in different stages using STEM software.

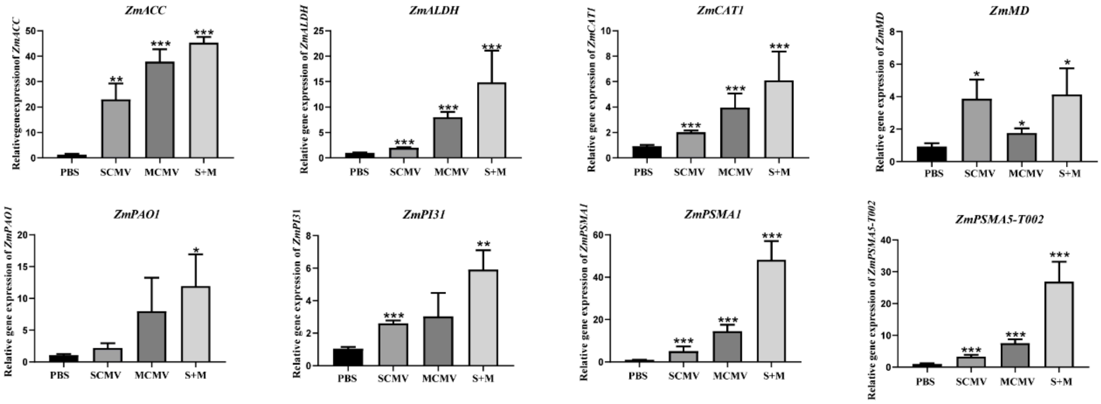

Figure S3: RT–qPCR-based validation of selected DEIs identified through RNA-Seq (n=3). The abbreviations of genes and compounds are explained in Table S10. Significance levels were set at * $p < 0.05$; ** $p < 0.01$; and *** $p < 0.001$.

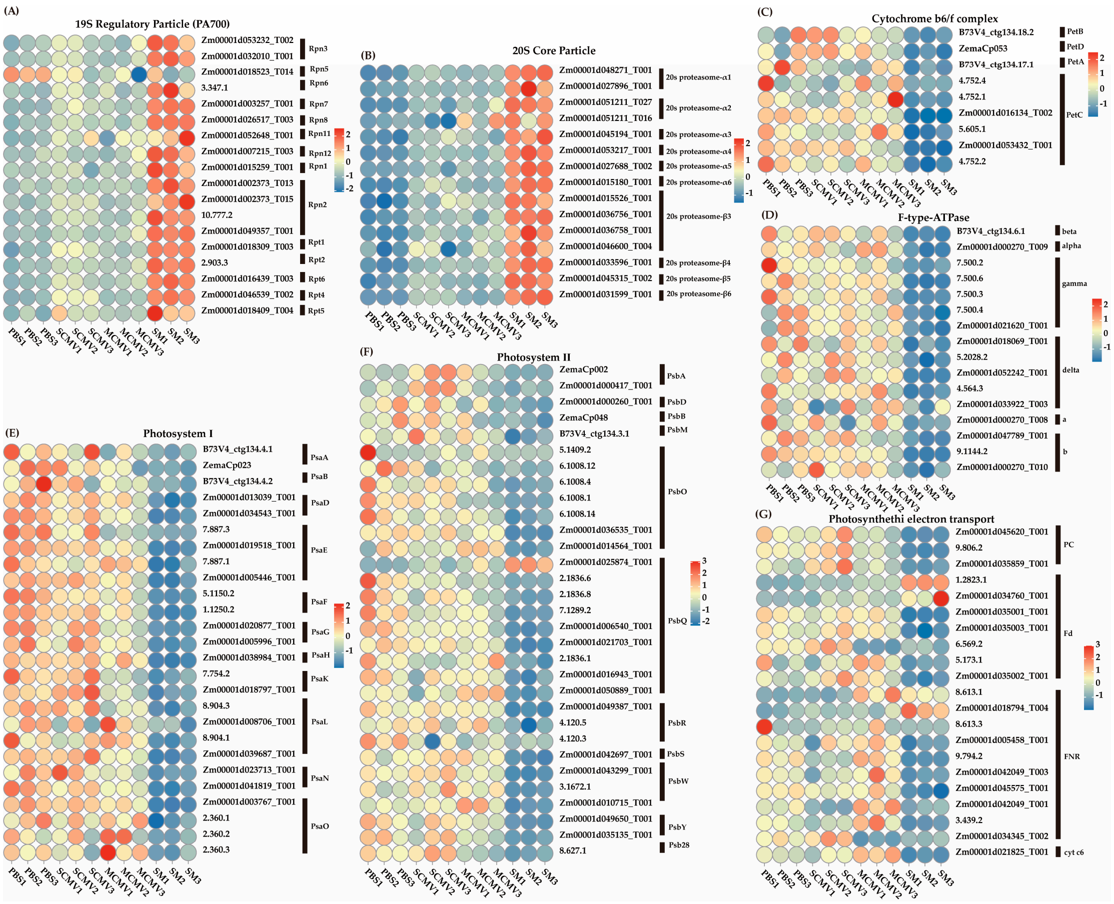

Figure S4: Heatmap of the expression levels of DEIs involved in different pathways in maize. (A) DEIs involved in the "19S Regulatory Particle (PA700)" of proteasome. (B) DEIs involved in the "20S Core Particle" of proteasome. (C) DEIs involved in "Cytochrome b6/f complex" of photosynthesis. (D) DEIs involved in "F-type-ATPase" of photosynthesis. (E) DEIs involved in "Photosystem I"of photosynthesis. (F) DEIs involved in "Photosystem II"of photosynthesis. (G) DEIs involved in "Photosynthethi electron transport" of photosynthesis.

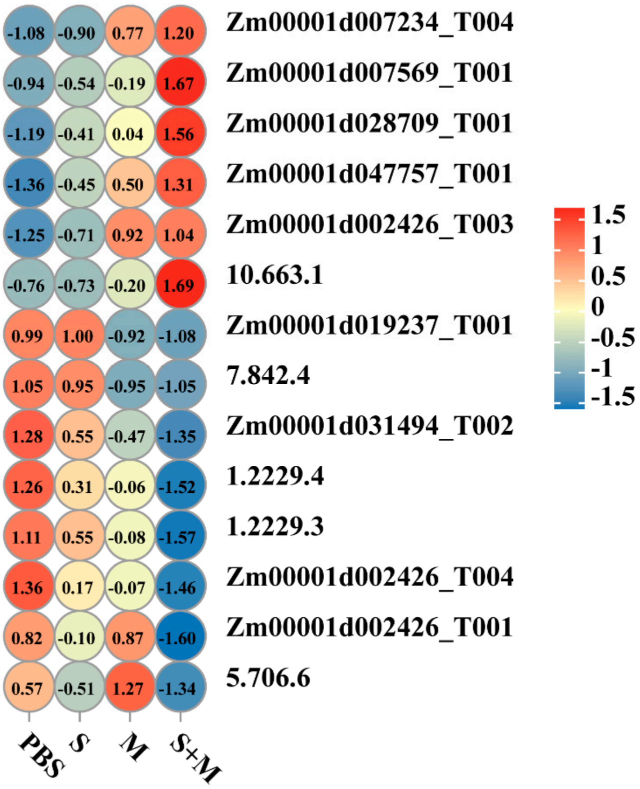

Figure S5: Heatmap of the expression levels of APXs.

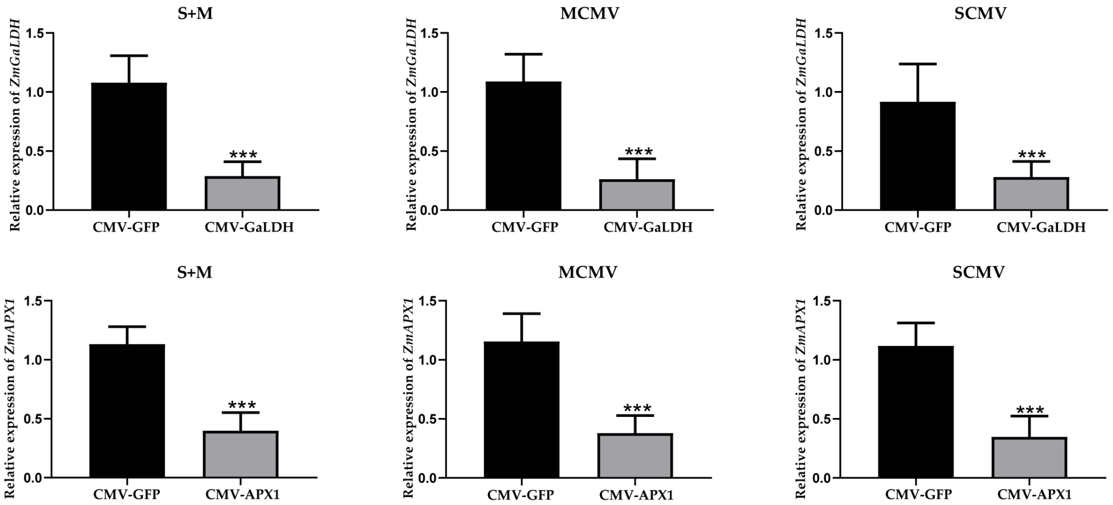

Figure S6: Silencing efficiencies of *ZmGalDH* or *ZmAPX1* through VIGS were determined through RT–qPCR. Significance levels were set at *** $p < 0.001$.

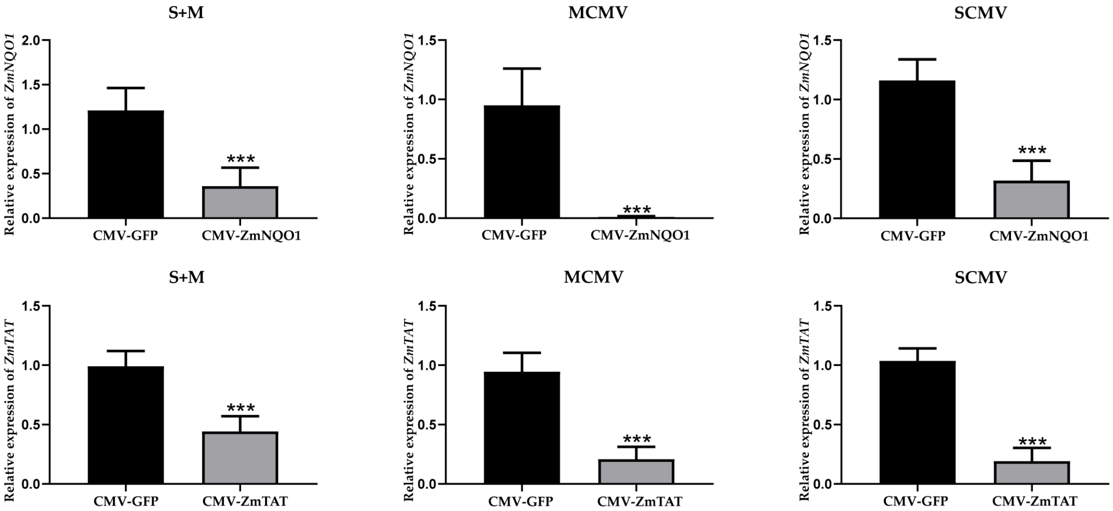

Figure S7: Silencing efficiencies of *ZmNQO1* or *ZmTAT* through VIGS were determined through RT–qPCR. Significance levels were set at *** $p < 0.001$.

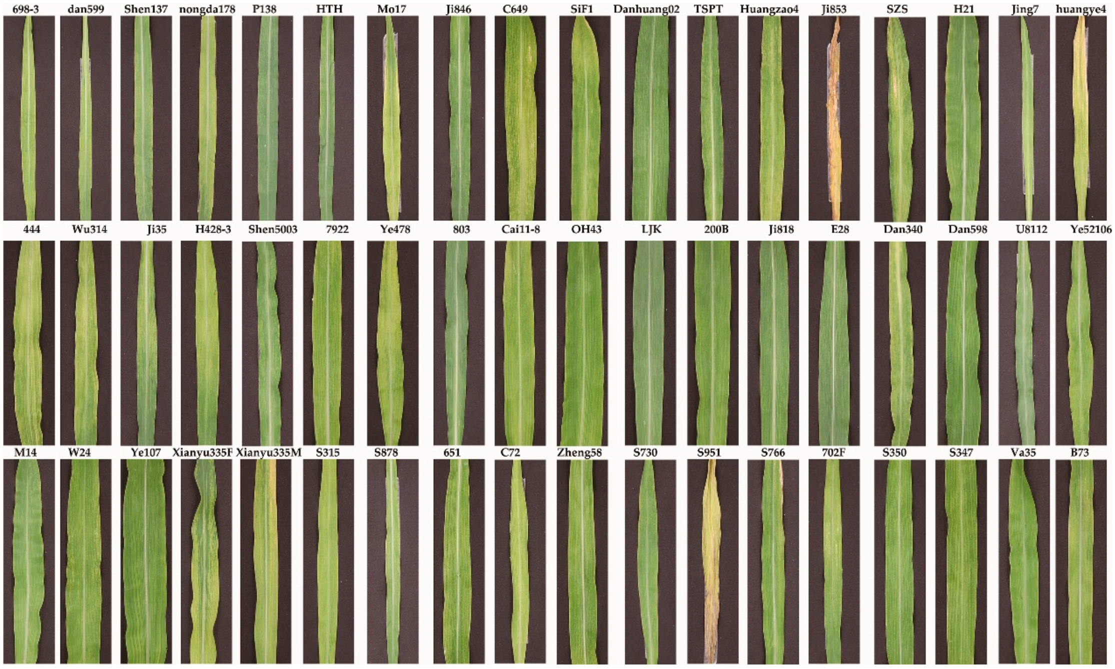

Figure S8: Resistance and susceptibility of 54 inbred lines to MCMV at 15 dpi.

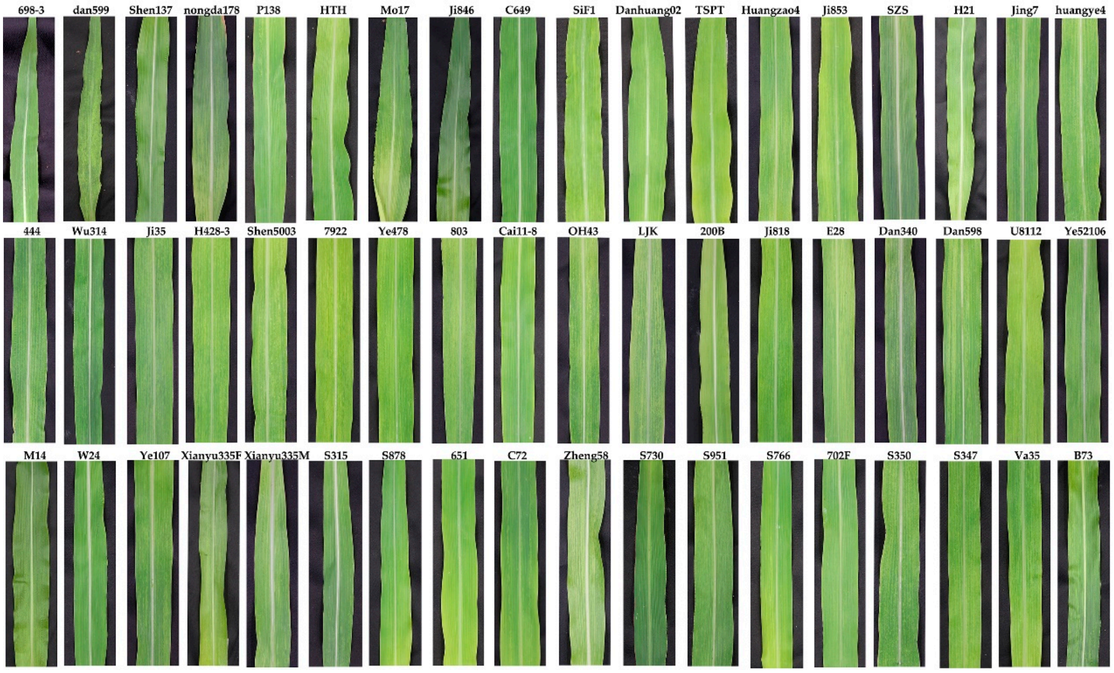

Figure S9: Resistance and susceptibility of 54 inbred lines to SCMV at 15 dpi.

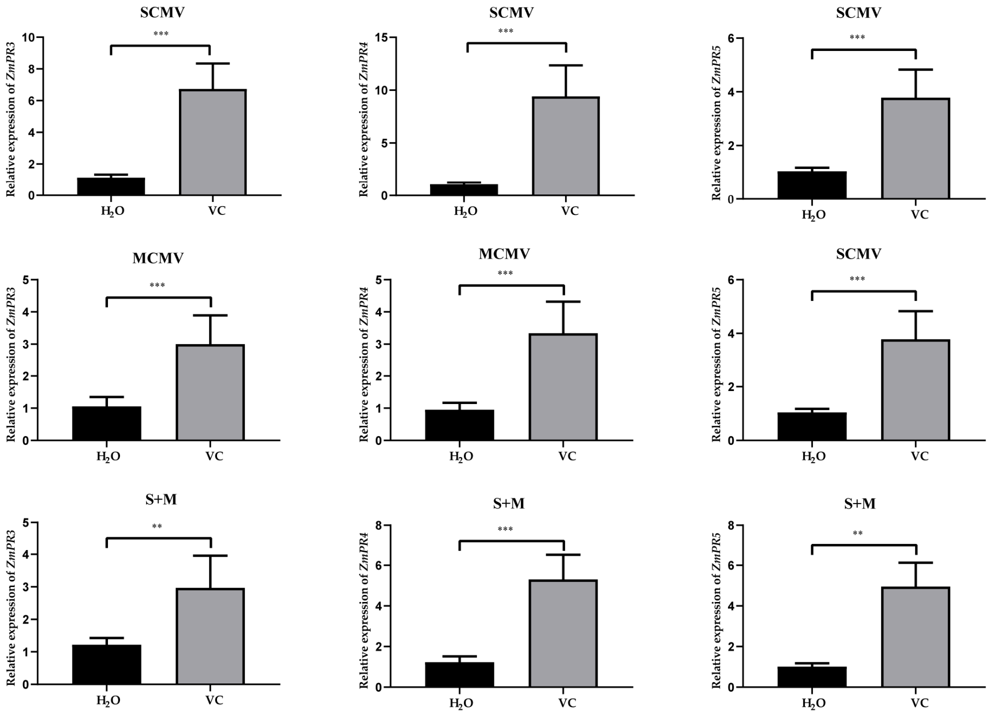

Figure S10: Analysis of SA-responsive *PR* gene expression in the leaves of maize plants or controls after being sprayed with vitamin C solutions. Vitamin C, AsA, VC. Significance levels were set at ** $p < 0.01$; and *** $p < 0.001$.

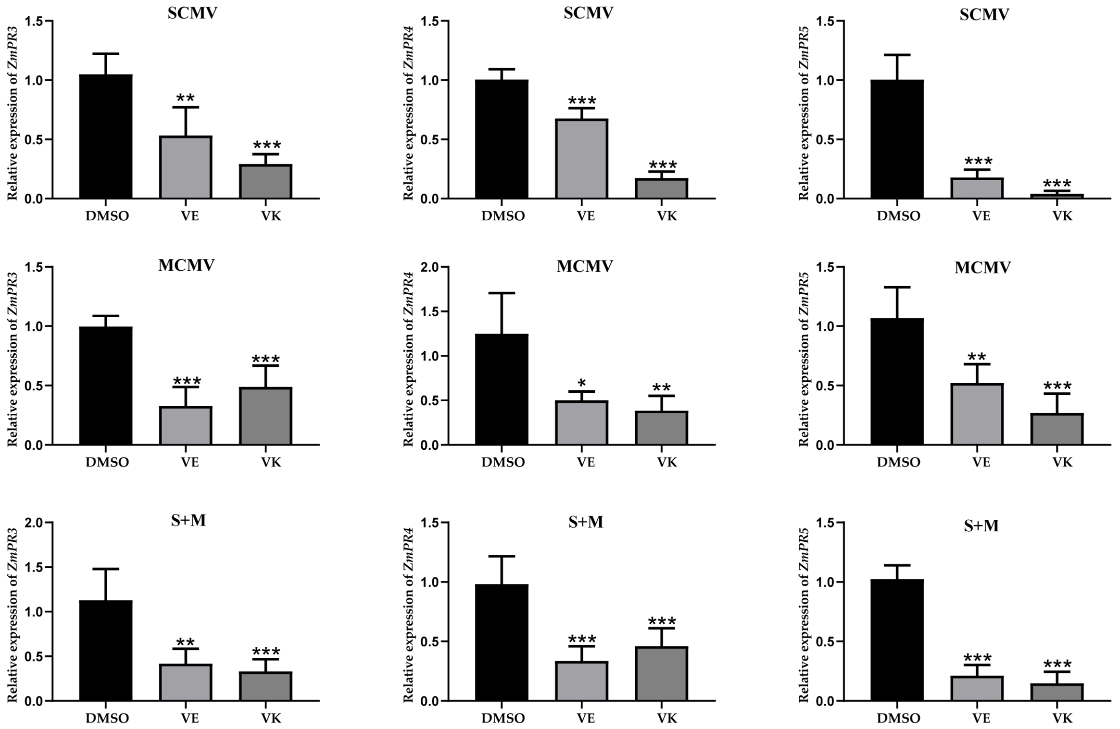

Figure S11: Analysis of SA-responsive *PR* gene expression in the leaves of maize plants or controls after spraying with vitamin E or K solution. Vitamin E, α-tocopherol, VE; vitamin K, vitamin K1, VK. Significance levels were set at * $p < 0.05$; ** $p < 0.01$; and *** $p < 0.001$.
